# Clinical characteristics of second primary pancreatic cancer

**DOI:** 10.1371/journal.pone.0179784

**Published:** 2017-06-26

**Authors:** Jung Hyun Jo, In Rae Cho, Jang Han Jung, Hee Seung Lee, Moon Jae Chung, Seungmin Bang, Seung Woo Park, Jae Bock Chung, Si Young Song, Jeong Youp Park

**Affiliations:** Division of Gastroenterology, Department of Internal Medicine, Severance Hospital, Yonsei University College of Medicine, Seoul, Korea; National Cancer Center, JAPAN

## Abstract

**Purpose:**

Several studies reported the increased risk of second primary pancreatic ductal adenocarcinoma (2nd PDAC) in cancer survivors. However, data on the characteristics of 2nd PDAC are insufficient.

**Methods:**

This retrospective cohort study included 1759 patients with PDAC. They were classified as having 2nd PDAC or first primary PDAC (1st PDAC) according to a prior diagnosed cancer of different origin, at least 6 months before PDAC diagnosis.

**Results:**

There were 110 patients (6.4%) with 2nd PDAC and 1606 (93.6%) patients with 1st PDAC. Patients with 2nd PDAC presented with older age (66.5 vs. 62.2 years, p < 0.001) and higher rate of resectability (26.4% vs. 15.9%, p = 0.004) at diagnosis than those with 1st PDAC. Multivariate analysis without considering resectable status showed that 2nd PDAC (hazard ratio [HR] 0.73, 95% confidence interval [CI] 0.56–0.94, p = 0.016) was associated with better overall survival. After adjusting for resectable status, however, 2nd PDAC (HR 0.85, 95% CI 0.66–1.09, p = 0.198) was no longer associated with overall survival. When subgroups were separately analyzed according to initial treatment modality, the effectiveness of surgery and chemotherapy were similar between 2nd and 1st PDAC (33.1 vs. 28.5 months, p = 0.860 and 10.8 vs. 10.7 months, p = 0.952).

**Conclusions:**

The proportion of resectable cases was significantly higher in 2nd PDAC. When surgery with curative aim was possible, the overall survival was increased even in patients with 2nd PDAC. These results suggest the importance of screening for second primary cancer in cancer survivors.

## Introduction

Second primary cancer is a new neoplasm that is biologically independent of a prior cancer.[1] The second primary cancer may share genetic factors or environmental background with the prior cancer, such as alcohol consumption, smoking, genetic syndromes/predisposition, and underlying diseases, which may be precancerous factors. Moreover, exposure to chemotherapy and radiation for prior cancer and receiving surgery can be predisposing factors to a second primary cancer. There were previous reports about an increased risk of secondary malignancies in cancer survivors.[2–4] The development of diagnostic and therapeutic techniques for malignancy has increased both the life span and the risk of second malignancies in cancer survivors. In the United States, the proportion of cancer survivors among the total population is estimated to be 3.5%, and about 10% of newly diagnosed cancers develop in cancer survivors.[5, 6] These data imply that understanding the characteristics of second primary cancers has become more important in developing screening and management programs for cancer survivors.

Pancreatic ductal adenocarcinoma (PDAC) is the fourth most common cause of cancer-related deaths and known to have an extremely poor prognosis, with a 5-year survival rate of <6%.[5] The median overall survival (OS) is 9 months for locally advanced pancreatic cancer and 3–6 months for metastatic disease.[7] The only potentially curative therapy for pancreatic cancer is surgical resection, and only 20% of pancreatic cancers are resectable at the time of diagnosis. In addition to this dismal prognosis, to date, there is no effective screening tool for high-risk patients and early screening for PDAC is not recommended for the general population in terms of cost-effectiveness.[8]

Similar to patients with other second primary malignancies, there have been patients with PDAC who developed another primary tumor in another organ or with a different histology. To date, there are only a few studies about second primary PDAC (2nd PDAC). A study about 2nd PDAC with pooled analysis of international multicenter cancer registries reported that the prevalence of 2nd PDAC among all patients with PDAC was about 6.3%.[9] There have been a few studies which reported pancreatic cancer as a second primary malignancy, and observed that the risk of second primary pancreatic cancer was increased after the development of various malignancies.[10] Recently, a report about second primary pancreatic cancer in colorectal cancer survivors showed that these patients have an increased risk of developing second primary pancreatic cancer.[11] However, although several studies have reported the increased risk of 2nd PDAC in cancer survivors, data on the characteristics of 2nd PDAC are insufficient. Thus, we conducted a study on the characteristics of 2nd PDAC, with the premise that our results might help in developing follow-up and screening strategies for cancer survivors, and in elucidating the treatment for these patients with fatal PDAC.

## Materials and methods

### Study population and definitions

A retrospective patient cohort from Severance Hospital, Seoul, Korea, was examined. A total of 1759 patients with PDAC, diagnosed as having ductal adenocarcinoma of the pancreas according to histopathological findings between 2006 and 2014, were included in this study. The age of the participants ranged from 44 to 91 years old.

The patients in the cohort were classified as having 2nd PDAC or first primary PDAC (1st PDAC) according to the history of a prior cancer. Patients who met the following criteria were considered as having 2nd PDAC: 1) the histological type of the prior cancer was not adenocarcinoma; 2) if the histological type of the previous tumor was adenocarcinoma, the possibility of primary pancreatic cancer should be higher than that of metastasis or recurrence of the previous tumor based on specific immunohistochemical staining or a reliable review by an experienced pathologist; and 3) PDAC was diagnosed at least 6 months after the diagnosis of the prior cancer to exclude synchronous PDAC. If the patient had a history of more than one prior cancer, the time of diagnosis of the first cancer was applied into the criteria.

The following clinical characteristics of patients were collected: sex, age, smoking status, alcohol consumption, history of diabetes and hypertension, CA19-9 level at diagnosis, primary location of pancreatic cancer, resectability at diagnosis, initial treatment modality, origin of another primary tumor, period between the time of diagnosis of another primary tumor and that of the 2nd PDAC, and OS.

### Statistical analysis

Data were analyzed by using the χ^2^, Fisher’s exact, and Student’s t-tests to compare variables between the groups, and a p-value of <0.05 was considered statistically significant. The OS and cumulative incidence rate of the 2nd PDAC after the diagnosis of other tumors were calculated and analyzed by using Kaplan-Meier analysis with a log-rank test. Hazard ratios (HRs), 95% confidence intervals (95% CIs), and p-values were calculated with a univariate Cox proportional hazards model for OS, by using the variables that were statistically significant. A multivariate Cox proportional hazards model was built to evaluate possible significant factors, taking into account the possible influence of confounding clinical variables. Only those variables that were significant in univariate analysis were entered into the multivariate analysis. Statistical analyses were carried out with Predictive Analytics Software statistics version 23.

### Ethical review

All procedures performed in studies involving human participants were in accordance with the ethical standards of the institutional research committee and with the 1964 Helsinki declaration and its later amendments or comparable ethical standards. As a retrospective analysis, Yonsei University Health System, Severance Hospital, Institutional Review Board approval was obtained and the need for informed consent was waived.

## Results

### Baseline characteristics

A total of 1759 patients with PDAC were included in the cohort. Forty-three patients were classified as having synchronous 2nd PDAC and excluded from the analysis. There were 1606 patients (93.6%) with 1st PDAC and 110 patients (6.4%) with 2nd PDAC in the study population (Table 1). Six patients (0.4%) had more than one prior cancer. In the comparison of baseline characteristics between the 1st PDAC and 2nd PDAC groups (Table 1), patients with 2nd PDAC presented significantly older age at diagnosis (66.5 vs. 62.2 years, p < 0.001), lower rate of alcohol consumption (25.5 vs. 36.8%, p = 0.017), higher rate of resectability of PDAC (26.4 vs. 15.9%, p = 0.004), and higher rate of receiving surgery as the initial treatment (26.4 vs. 15.9%, p = 0.018) than patients with 1st PDAC. The origins of prior cancers accompanied with 2nd PDAC are summarized in Table 2. The most common origin of prior cancers was the stomach (22 of 110, 20.0%), followed by the thyroid (21 of 110, 19.1%), breast (19 of 110, 17.3%), colon (12 of 110, 10.9%), and others.

**Table 1:** Baseline characteristics of subgroups.

	Total	1st PDAC	2nd PDAC	P-value
Patients	1716 (100%)	1606 (93.6%)	110 (6.4%)	
Age, mean (SD)	62.5 (±10.3)	62.2 (±10.3)	66.5 (±9.6)	<0.001
Sex				0.335
Women	705 (41.1%)	655 (40.8%)	50 (45.5%)	
Men	1011 (63.9%)	951 (59.2%)	60 (54.5%)	
Alcohol				0.017
Yes	619 (36.1%)	591 (36.8%)	28 (25.5%)	
No	1097 (63.9%)	1015 (63.2%)	82 (74.5%)	
Smoking				0.067
Yes	557 (32.5%)	530 (33.0%)	27 (24.5%)	
No	1159 (67.5%)	1076 (67.0%)	83 (75.5%)	
Diabetes				0.817
Yes	560 (32.6%)	523 (32.6%)	37 (33.6%)	
No	1156 (67.4%)	1083 (67.4%)	73 (66.4%)	
Hypertension				0.940
Yes	661 (38.5%)	619 (38.5%)	42 (38.2%)	
No	1055 (61.5%)	987 (61.5%)	68 (61.8%)	
Initial CA19-9 (U/mL)	3591.5 (±6336.9)	3620.8 (±6377.5)	3169.0(±5730.9)	0.474
Location of main mass				0.219
Head	774 45.1%)	727 (45.3%)	47 (42.7%)	
Body	394 (23.0%)	361 (22.5%)	33 (30.0%)	
Tail	315 (18.4%)	295 (18.4%)	20 (18.2%)	
Overlap	233 (13.6%)	223 (13.9%)	10 (9.1%)	
Resectability				0.004
Resectable	285 (16.6%)	256 (15.9%)	29 (26.4%)	
Unresectable	1431 (83.45%)	1350 (84.1%)	81 (73.6%)	
Initial treatment				0.018
Surgery	285 (16.6%)	256 (15.9%)	29 (26.4%)	
Chemotherapy	1160 (67.6%)	1094 (68.1%)	66 (60.0%)	
No treatment	271 (15.8%)	256 (15.9%)	15 (13.6%)	
Median OS, months	11.8 (0.0–100.6)	11.8 (0.0–100.6)	12.3 (0.3–86.5)	0.068

*Abbreviations*: PDAC, pancreatic ductal adenocarcinoma; SD, standard deviation; OS, overall survival.

**Table 2:** Origins of prior cancers accompanied with second primary PDAC.

Other cancer origins	Patient number	Interval since prior cancer, years (range)
Total	110 (100%)	8.4 (0.7–31.4)
Stomach	22 (20.0%)	11.2 (1.3–23.2)
Thyroid	21 (19.1%)	5.0 (1.2–30.5)
Breast	19 (17.3%)	9.5 (1.8–30.5)
Colon	12 (10.9%)	5.3 (0.7–26.8)
Prostate	5 (4.5%)	2.9 (0.8–10.0)
Rectum	5 (4.5%)	9.3 (3.0–14.2)
Head and neck	4 (3.6%)	9.0 (2.7–17.3)
Bile duct	3 (2.7%)	6.5 (5.8–11.6)
Cervix	3 (2.7%)	19.2 (17.3–31.4)
Bladder	3 (2.7%)	2.1 (1.2–13.1)
Lung	2 (1.8%)	10.6 (5.3–15.9)
Ovary	2 (1.8%)	9.8 (9.5–10.0)
Hematologic malignancy*	2 (1.8%)	4.3 (2.6–6.1)
Liver	1 (0.9%)	1.6 (1.6–1.6)
Kidney	1 (0.9%)	14.8 (14.8–14.8)
Esophagus	1 (0.9%)	4.5 (4.5–4.5)
AOV	1 (0.9%)	9.1 (9.1–9.1)
Endometrium	1 (0.9%)	12.8 (12.8–12.8)
Brain	1 (0.9%)	7.5 (7.5–7.5)
Other^†^	1 (0.9%)	10.7 (10.7–10.7)

*Hematologic malignancy: one multiple myeloma and one aplastic large-cell lymphoma.

^†^Other: a paratesticular tumor (soft tissue cancer).

*Abbreviations*: PDAC, pancreatic ductal adenocarcinoma; AOV, ampulla of Vater.

### Survival analysis

The OS was slightly longer in patients with 2nd PDAC; however, the difference was not significant (11.8 vs. 12.3 months, p = 0.068). Multivariate analysis without resectable status showed that 2nd PDAC (HR 0.73, 95% CI 0.56–0.94, p = 0.016), age at diagnosis (HR 1.02, 95% CI 1.01–1.02, p < 0.001), and alcohol consumption (HR 1.28, 95% CI 1.13–1.47, p < 0.001) were significantly related to OS (Table 3). When resectable status was included in multivariate analysis, age at diagnosis (HR 1.02, 95% CI 1.01–1.02, p < 0.001), alcohol consumption (HR 1.25, 95% CI 1.11–1.42, p = 0.001), and resectable status at diagnosis (HR 0.30, 95% CI 0.25–0.36, p < 0.001) were significantly associated with OS. However, 2nd PDAC (HR 0.85, 95% CI 0.66–1.09, p = 0.198) was no longer significantly associated with OS after adjusting for resectable status. This analysis suggested that the association between 2nd PDAC and survival was owing to the higher resectability rate.

**Table 3:** Cox proportional analysis for the contribution of clinical factors to overall survival.

	Univariate	Multivariate		Multivariate*	
	P-value	HR (95% CI)	P-value	HR (95% CI)	P-value
Second PDAC	0.093	0.73 (0.56–0.94)	0.016	0.85 (0.66–1.09)	0.198
Older age	<0.001	1.02 (1.01–1.02)	<0.001	1.02(1.01–1.02)	<0.001
Male sex	0.056	1.04 (0.90–1.19)	0.627	1.03(0.90–1.19)	0.645
Alcohol use (vs. non-alcohol use)	0.001	1.28 (1.13–1.47)	<0.001	1.25 (1.11–1.42)	<0.001
Resectable disease (vs. unresectable)	<0.001	Not included		0.30 (0.25–0.36)	<0.001

*Abbreviations*: PDAC, pancreatic ductal adenocarcinoma; SD, standard deviation; HR, hazard ratio; CI, confidence interval.

* Multivariate analysis including resectable disease and multivariate analysis without resectable disease are separately presented.

**Fig 1:**
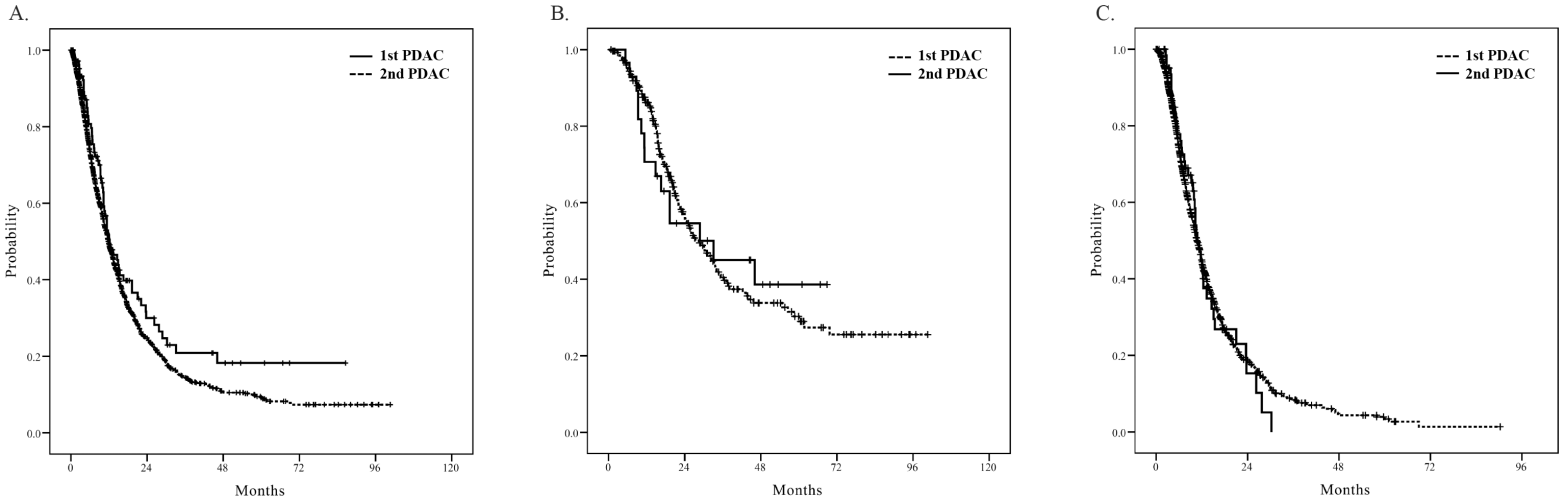
Kaplan-Meier analysis of overall survival (OS) according to subgroup. (A) First primary pancreatic ductal adenocarcinoma (1st PDAC) vs. second primary PDAC (2nd PDAC) in the whole cohort (n: 1606 vs. 110): median OS 11.8 vs. 12.3 months, p = 0.068 by log-rank test. (B) 1st PDAC vs. 2nd PDAC in patients who received curative surgery as initial treatment (n: 256 vs. 29): median OS 28.5 vs. 33.1 months, p = 0.860 by log-rank test. (C) 1st PDAC vs. 2nd PDAC in patients who received chemotherapy as initial treatment (n: 1094 vs. 66): median OS 10.7 vs. 10.8 months, p = 0.952 by log-rank test.

To exclude the confounding effect of the resectability of PDAC on OS, we divided the whole cohort according to the initial treatment modality for PDAC: 1) patients who received curative surgery and 2) patients who received chemotherapy. Then, the OS of patients with 1st PDAC and those with 2nd PDAC was compared with Kaplan-Meier analysis according to treatment modality (Fig 1). In the subgroup of patients who received curative surgery (Fig 1B), the median OS was 28.5 months (95% CI, 23.0–34.1) in the 1st PDAC group compared with 33.1 months (95% CI, 9.0–27.2) in the 2nd PDAC group (n: 259 vs. 29, p = 0.860). In the subgroup of patients who received chemotherapy (Fig 1C), the median OS was 10.7 months (95% CI, 10.0–11.4) in 1st PDAC compared with 10.8 months (95% CI, 9.2–12.3) in 2nd PDAC (n: 1094 vs. 66, p = 0.952). The effectiveness of either surgery or chemotherapy was similar between the 2nd and 1st PDAC groups.

To compare differences according to the follow-up modality that might be different depending on the location of the prior cancer, we divided patients with 2nd PDAC into those with a prior cancer originating from the abdomen and those with prior cancer of non-abdominal origin (Table 4). The origin of abdominal prior cancers included the gastrointestinal tract (stomach, colon, rectum), hepatobiliary tract (liver, bile duct), genitourinary tract (prostate, bladder, kidney), gynecologic system (cervix, endometrium, and ovary), and other sites in the abdominal and pelvic cavities. Other cancers originating from the thyroid, breast, head and neck, lung, and brain were included in the non-abdominal prior cancer group. There were 63 patients (57.2%) with abdominal prior cancer and 47 patients (42.7%) with non-abdominal prior cancer. In the comparison analysis, there were no significant differences in resectability (27.0 vs. 25.5%, p = 0.864) or OS (11.3 vs. 14.5 months, p = 0.212) between groups.

**Table 4:** Comparison of subgroups according to the location of the prior cancer in second PDAC.

	Abdomen	Non-abdomen	P-value
Patients	63 (57.2%)	47 (42.7%)	
Age, mean (SD)	67.8 (±9.7)	64.7 (±9.4)	0.087
Sex			<0.001
Women	16 (25.4%)	34 (72.3%)	
Men	47 (74.6%)	13 (27.7%)	
Interval from prior cancer	8.9 (0.7–31.4)	8.4 (1.2–30.5)	0.766
Alcohol	18 (28.6%)	10 (21.3%)	0.385
Smoking	20 (31.7%)	7 (14.9%)	0.042
Diabetes	23 (36.5%)	14 (29.8%)	0.461
Hypertension	40 (63.5%)	28 (59.6%)	0.676
Initial CA19-9 (U/mL)	2957.0 (±5596.2)	3454.8 (±5957.8)	0.657
Location of main mass			0.553
Head	24 (38.1%)	23 (48.9%)	
Body	19 (30.2%)	14 (29.8%)	
Tail	14 (22.2%)	6 (12.8%)	
Overlap	6 (9.5%)	4 (8.5%)	
Resectability			0.864
Resectable	17 (27.0%)	12 (25.5%)	
Unresectable	46 (73.0%)	35 (74.5%)	
Initial treatment			0.352
Surgery	17 (27.0%)	12 (24.6%)	
Chemotherapy	35 (55.6%)	31 (66.0%)	
No treatment	11 (17.5%)	4 (8.5%)	
Median OS, months	11.3 (0.3–86.5)	14.5 (0.4–68.9)	0.212

*Abbreviations*: SD, standard deviation; OS, overall survival.

In addition, we analyzed ratios of incidental findings on abdominal follow-up diagnostics in comparison to symptomatic 2nd primary PDAC, which led to scan-diagnostics in the first place. There were 47 patients who were diagnosed with 2nd PDAC on follow-up abdominal CT/MRI scans and 63 patients who were diagnosed with 2nd PDAC by symptoms. Comparing the two groups, the rate of resectable disease was significantly higher in the patients who were diagnosed by follow-up abdominal CT/MRI scans than those diagnosed by symptoms presentation (36.2% vs. 19.0%, p = 0.044).

### Cumulative incidence rate of 2nd PDAC after diagnosis of another primary tumor

**Fig 2:**
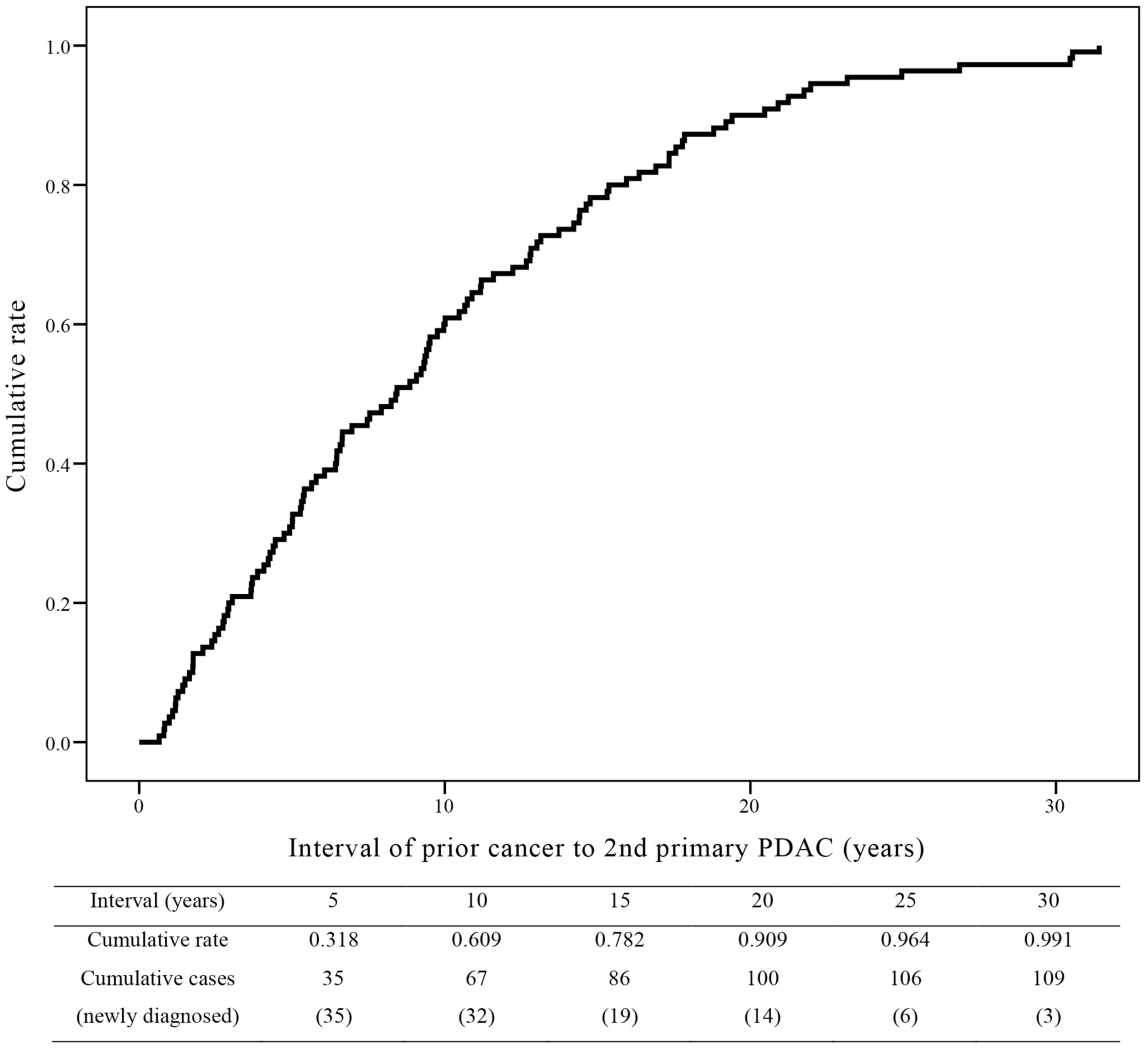
Cumulative curve of diagnosis of second pancreatic ductal adenocarcinoma (PDAC). Cumulative incidence rate of second primary PDAC (2nd PDAC) after the diagnosis of prior cancer in the 2nd PDAC group. The median interval between the diagnosis of the 2nd PDAC and the diagnosis of the prior cancer was 8.4 years. The cumulative rates (cumulative cases for every 5 years after the diagnosis of prior cancer) are presented as a table.

The median interval between the diagnosis of the 2nd PDAC and the diagnosis of the prior cancer was 8.4 years (range 0.7–31.4 years) in the 2nd PDAC group (Table 2). Among prior cancers with a ratio of >10%, the median interval of stomach cancer was the longest at 11.2 years (range 1.3–23.2 years). Thyroid cancer presented the shortest interval to 2nd PDAC at 5.0 years (range 1.2–30.5 years). Fig 2 shows the cumulative incidence rate of 2nd PDAC after the diagnosis of prior cancer in the 2nd PDAC group. The trend of the first 5 years was 7 cases per year (35 cases every 5 years). Thereafter, every 5 years, the value decreased continuously to 6.4, 3.8, 2.8, 1.2, and 0.6 cases per year until 30 years. The cumulative incidence rate was 60.9% at 10 years, 90.9% at 20 years, and 99.1% at 30 years after the diagnosis of prior cancer.

## Discussion

Previously, there were a few studies about 2nd PDAC with a population-based analysis. These studies reported that cancer survivors have an increased risk of developing 2nd PDAC.[10, 12, 13] Although there have been reports about the increased risk of 2nd PDAC in cancer survivors, there have been few studies about how the 2nd PDAC was different from the 1st PDAC. Hackert *et al* reported features of patients with PDAC and history of extrapancreatic malignancy compared to patients with PDAC as only tumor, however, the results of the study were limited to patients who underwent surgery for PDAC.[14] Considering the fatality of pancreatic cancer, an increased risk of pancreatic cancer in cancer survivors necessitates more attention on 2nd PDAC. In that context, we aimed to analyze the characteristics of 2nd PDAC in this study.

In this study, there were 110 patients with 2nd PDAC among a total of 1716 patients with PDAC. The proportion of patients with 2nd PDAC was about 6.4%, and this percentage was compatible with that reported in a previous study.[9] The considerable proportion of patients with 2nd PDAC in the entire pancreatic cohort again suggests that more attention should be paid to this specific subset of patients with pancreatic cancer.

In our results, the patient age at diagnosis of 2nd PDAC was older than that of 1st PDAC (66.5 vs. 62.2 years, p < 0.001). Similarly, in a previous report, there were more patients >75 years old (46% vs. 32%) and fewer patients <65 years old (22% vs. 37%) in the 2nd PDAC group than in the 1st PDAC group.[9] This result concurs with another study which reported that patients with PDAC and history of extrapancreatic neoplasm presented at older age than patients with PDAC as only tumor in patients who underwent surgery (68 vs. 64 years, p < 0.0001).[14] There seemed to be a tendency that patients with second primary cancer are older than patients with first primary cancer at the time of diagnosis. A previous study about second primary non-small cell lung cancer (NSCLC) reported that the group of patients with NSCLC with a history of another tumor presented older age at diagnosis (71 vs. 66 years) than did patients with NSCLC as the only tumor, similar to our result.[15] The age at diagnosis might be different because old age is one of the risk factors of cancer and patients with second primary cancer had lived long enough to survive the prior cancer.

The rate of alcohol consumption (25.5 vs. 36.8%, p = 0.017) was lower in the 2nd PDAC group in our results. This might be because of the tendency of patients to stop drinking alcohol upon the diagnosis of cancer. Like our study, a previous study reported that the onset of cancer was significantly associated with cessation of alcohol consumption, based on cohort data from 9001 Korean patients.[16]

In the multivariate analysis after adjusting for confounding factors such as older age at diagnosis of 2nd PDAC, 2nd PDAC was significantly associated with a longer OS compared with 1st PDAC (HR 0.73, p = 0.016). However, after adjusting for resectability at diagnosis, 2nd PDAC was no longer significantly associated with OS. In fact, the rate of resectability of 2nd PDAC was significantly higher than that of 1st PDAC (26.4 vs. 15.9%, p = 0.004). Furthermore, more patients with 2nd PDAC could receive curative surgery, which could potentially increase their OS. When the whole cohort was divided into subgroups according to initial treatment modality (curative surgery or chemotherapy), there was no difference in OS between 2nd PDAC and 1st PDAC in both subgroups. These results suggest that the OS of the 2nd PDAC group seemed to be longer because there were more resectable cases at the time of diagnosis. Concerning the treatment, it can be presumed that that the nature of 2nd PDAC and 1st PDAC was not much different. When surgery of curative aim was possible, the OS was increased even in patients with 2nd PDAC. This result suggests the need to develop screening programs for detecting resectable pancreatic cancer in cancer survivors.

To identify the effect of follow-up methods that might be different depending on the anatomical distribution of the prior cancer, we compared the subgroups divided according to the location of prior cancer in the 2nd PDAC group. When comparing cancers of abdominal origin, which were closer to the pancreas, and those of non-abdominal origin, there were no significant differences between subgroups in terms of resectability (p = 0.864) or OS (p = 0.212). It seems that the origin of prior cancer was not related to the tendency of early detection of 2nd PDAC. Interestingly, the rate of resectable disease was significantly higher in the patients who were diagnosed by follow-up abdominal CT/MRI scans than those diagnosed by presentation of symptomatic 2nd PDAC, which led to scan-diagnostics in the first place. It seemed that there was a correlation between early detection and follow-up methods, which suggested the necessity of developing screening programs for 2nd PDAC. However, there were only 110 patients with 2nd PDAC in this study, and follow-up intervals and methods were heterogeneous. A larger study with subgroup analysis according to the cell types or common etiologies shared by prior cancers should be conducted in the future. Most importantly, an optimal follow-up program for 2nd PDAC should be developed and evaluated.

The cumulative curve of diagnosis of 2nd PDAC is presented in Fig 2. According to our analysis, the median interval from prior cancer to 2nd PDAC was 8.4 years, and the cumulative rate of diagnosis of 2nd PDAC continuously increased for 10 years with inclination similar to the first 5 years (6.4 and 5.8 cases per year). The graph was steadily increasing for 20 years with a 90.9% cumulative rate and then reached a plateau. This result implies that cancer screening in cancer survivors and regular follow-up need to continue up to 20 years after the diagnosis of prior cancer, which is longer than the usual current follow-up period for most cancers. In our study, the most common prior cancer of 2nd primary PDAC was stomach cancer (20.0%), followed by thyroid cancer (19.1%), breast cancer (17.3%), colon cancer (10.9%), and others. This result was compatible with the prevalence and OS of these cancers in Korea. According to Cancer Statistics 2013 in Korea,[17] the most common origin of cancer was the stomach (17.8%) and colon (14.6%) in men. In women, thyroid cancer (30.5%) was the most common and breast cancer (15.4%) was the second common malignancy. In our study, there were only a few patients with lung cancer or liver cancer as the prior cancer, although these cancers are highly prevalent in Korea. This result was presumed to be because of the poor survival of patients with liver and lung cancers.

This study has several limitations. The number of patients with 2nd PDAC was small compared with those with 1st PDAC, although the proportion of 2nd PDAC in the total cases of PDAC was compatible with a previous study. In addition, we did not consider the possibility of multicancer syndromes accompanied with genetic disorders and the presence of premalignant lesions of PDAC such as intraductal papillary mucinous tumor. To overcome such limitations, larger prospective studies are needed in the future.

## Conclusions

Second primary pancreatic cancer had a higher rate of resectability, and there was no difference in the effectiveness of curative surgery and chemotherapy between 2nd and 1st PDAC. Therefore, when curative surgery for 2nd PDAC is possible, it should be conducted similarly to curative surgery for 1st PDAC. Considering the increased risk of 2nd PDAC in cancer survivors and the fact that surgery is the only curative treatment for this fatal cancer, more efforts are needed to develop screening programs for second primary pancreatic cancer in cancer survivors.
